# An Unusual Lipoma at the Lateral Margin of the Tongue: A Case Report

**DOI:** 10.1155/crid/1266010

**Published:** 2026-07-19

**Authors:** Guojun Yang, Zhen Liu, Jingbiao Wu, Ming Zhang, Binbin Jing, Qiao Li

**Affiliations:** ^1^ Department of Stomatology, Affiliated Hospital of North Sichuan Medical College, Nanchong, Sichuan Province, China, hospital-nsmc.com.cn; ^2^ Department of Oral and Maxillofacial Surgery, Affiliated Hospital of North Sichuan Medical College, Nanchong, Sichuan Province, China, hospital-nsmc.com.cn

**Keywords:** case report, CT, lingual, lipoma, minimally invasive resection, tongue neoplasms

## Abstract

Lipoma is a common benign mesenchymal tumor, but oral lipomas account for only 1%–4% of all lipomas, with lingual lateral margin lipomas being an even rarer subtype. A 53‐year‐old female presented with a 1‐year history of a slow‐growing, painless, and pale yellow submucosal mass on the left lateral tongue (1.5‐cm in diameter). Preoperative contrast‐enhanced maxillofacial CT suggested a cystic space‐occupying lesion, and minimally invasive resection was performed under local anesthesia. Histopathological examination confirmed a classic lipoma with mature, uniformly arranged adipocytes and no atypia. The patient had uneventful wound healing, no postoperative complications, and no recurrence at 3‐week follow‐up, with full recovery of tongue function. Literature review indicated that contrast‐enhanced CT combined with typical clinical signs improves preoperative diagnostic accuracy, and minimally invasive local resection is a safe, effective treatment for small lingual lipomas. This case adds new clinical evidence for small lipomas of the lateral tongue and improves the dataset of this rare tumor subtype, offering guidance for clinical practice. This report also reminds clinicians that soft yellow nodules on the tongue margin cannot be simply diagnosed as cysts according to CT hypodensity alone. Combined clinical and radiological assessment is mandatory, and local minimally invasive surgery is the optimal option for these small lesions.

## 1. Introduction

Lipoma is a common benign mesenchymal tumor composed of mature adipocytes, characterized by slow growth, well‐defined borders, and an intact capsule [1]. Systemically, it is classified into superficial subcutaneous, subfascial deep, and intramuscular subtypes, with the latter being rare [2]. The World Health Organization (WHO) categorizes benign lipomas into 11 subtypes, providing a basis for clinical differential diagnosis [3, 4]. Lipomas require differentiation from epidermal cysts, hematomas, and malignant adipocytic tumors to avoid misdiagnosis [5].

Head and neck lipomas account for 20% of all lipomas [1, 6], whereas oral lipomas represent only 1%–4% [3, 7], with common sites including the buccal mucosa, lips, palate, and tongue. These lesions have an excellent prognosis with low recurrence after complete resection [5, 8–11]. Oral lipomas are typically slow‐growing, painless, asymptomatic masses with a mean diameter of ~2.0 cm [11], and large lesions may impair mastication and speech [12]. Their pathogenesis is unclear, with potential associations with trauma, chronic inflammation, and hormonal imbalance [13].

Lingual lipomas constitute a small proportion of all oral lipomas, and obvious anatomical differences exist among different lingual sites. Most lingual lipomas are found at the tongue base and ventral surface [14]. In contrast, lipomas originating from the lateral tongue margin are extremely rare, with limited clinical reports worldwide. Large‐sample statistical data demonstrate that lateral margin lipomas account for less than 2.8% of all lingual lipomas. Particularly, submucosal small lesions with a diameter less than 2 cm are scarcely documented, which are regarded as rare benign neoplasms in the oral region [14].

The present case involves a small lingual lateral margin lipoma measuring 1.5 cm in diameter, which possesses unique clinical characteristics. The lateral tongue features thin mucosa and sparse subcutaneous adipose tissue, lacking the anatomical fat pad that predisposes to lipoma formation [15]. Furthermore, small lipomas at this site share similar imaging density features with mucous cysts and epidermal cysts. Consequently, contrast‐enhanced CT examinations frequently misinterpret such solid lesions as cystic space‐occupying lesions preoperatively [16, 17], leading to a high rate of missed diagnosis and misdiagnosis. This also explains the scarcity of relevant clinical cases. Herein, we report the clinical manifestations, imaging findings, surgical procedures and histopathological features of this rare small lipoma of the lateral tongue margin, combined with a comprehensive literature review, to provide practical references for the diagnosis and standardized management of this uncommon oral tumor.

## 2. Case Presentation

### 2.1. Medical History

A 53‐year‐old female patient presented with a painless mass on the left lateral tongue that had gradually enlarged over the past year. One year prior to presentation, the patient incidentally noticed a mung bean‐sized, painless submucosal lesion at the left tongue margin. The mass grew slowly and progressively throughout the disease course, without local pain, pruritus, or ulceration. No limitation of tongue movement, masticatory disturbance, or speech impairment was observed. The patient had a generally good physical condition and denied a history of endocrine disorders, chronic inflammation, lingual, and maxillofacial trauma. There was no history of drug or food allergy. She had no habits of cigarette smoking or alcohol consumption. None of her immediate family members had a diagnosis of lipoma, lipid metabolism disorders, or soft tissue neoplasms. No genetic testing was performed, and no evidence indicated hereditary diseases. The patient had not received any targeted treatments, including medication, puncture, physical therapy, or surgical intervention at other medical institutions before admission.

### 2.2. Clinical Findings

**Figure 1:**
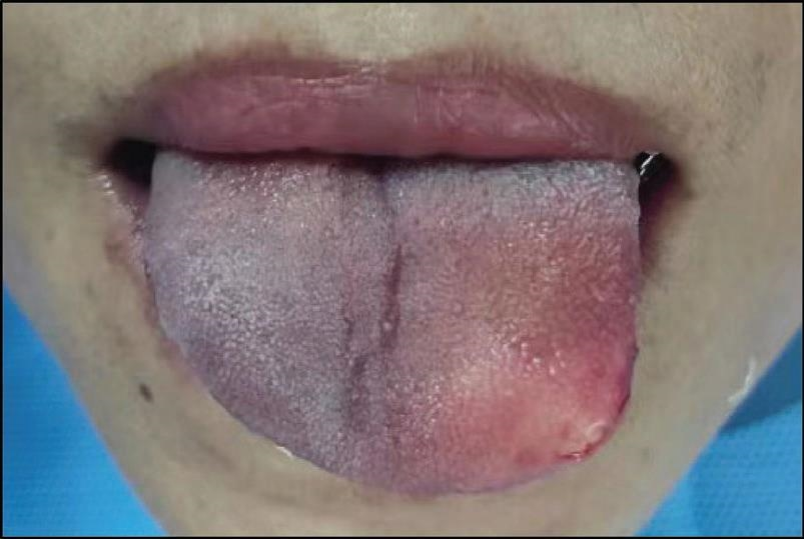
Clinical examination revealed a submucosal mass with a diameter of approximately 1.5 cm and yellowish color on the left lateral border of the tongue.

The patient exhibited normal mouth opening range and opening pattern. No abnormalities were found in the dentition and floor of the mouth. A pale yellow submucosal mass measuring approximately 1.5 cm in diameter was identified on the left lateral tongue (Figure 1). The mass was soft, freely mobile, nontender on palpation, and well‐demarcated from adjacent tissues without adhesion. Tongue movement, speech, taste, and sensation were intact, with no bilateral cervical lymphadenopathy.

### 2.3. Imaging Examination

**Figure 2:**
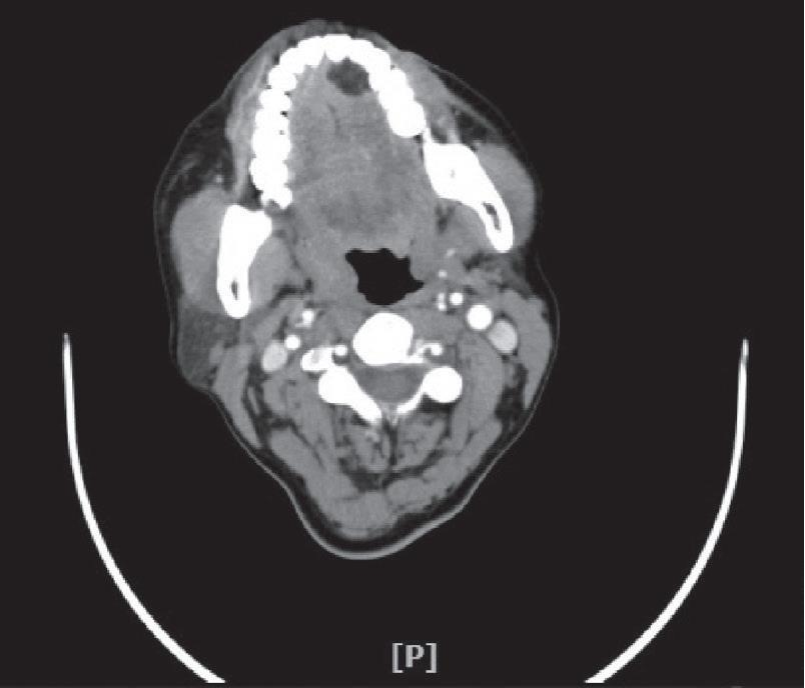
Contrast‐enhanced computed tomography (CT). A well‐circumscribed, oval, and low‐density lesion, with a few internal septations, approximately 1.5 cm in diameter, is appreciable on the left lateral border of the tongue.

Contrast‐enhanced maxillofacial CT showed an oval low‐density lesion (1.5 × 0.9 cm) in the left lateral border of tongue with clear boundaries, slight internal septa, and mild peripheral enhancement. No mandibular bone abnormalities were detected, and the CT report suggested a cyst‐predominant space‐occupying lesion requiring further clinical correlation (Figure 2).

### 2.4. Treatment Process

**Figure 3:**
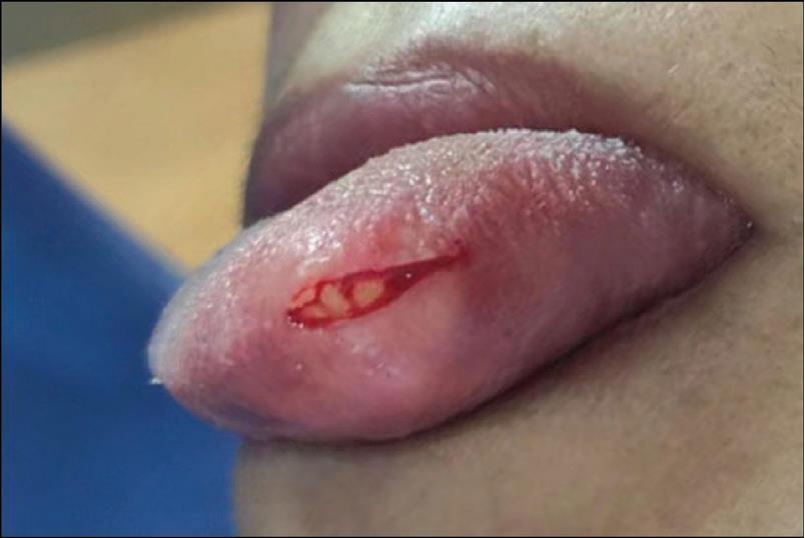
Upon surgical incision, a pale yellow mass was observed protruding beneath the lingual mucosa.

**Figure 4:**
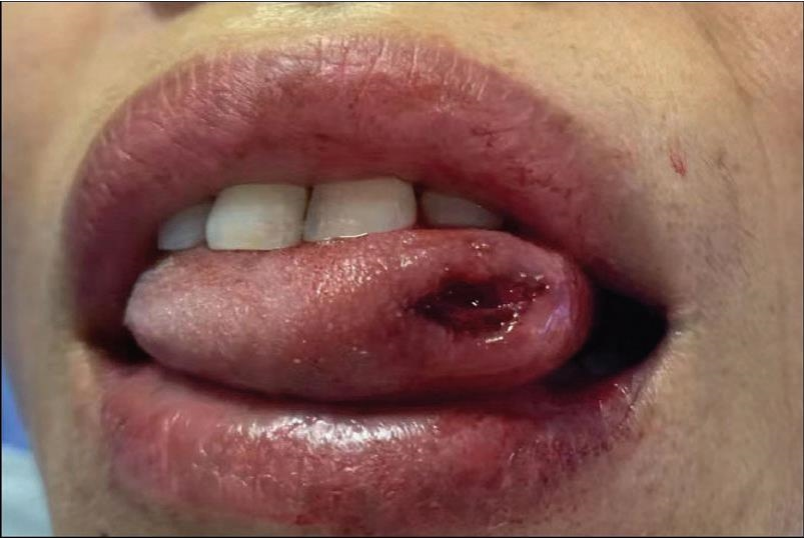
Surgical field after complete removal of the mass.

**Figure 5:**
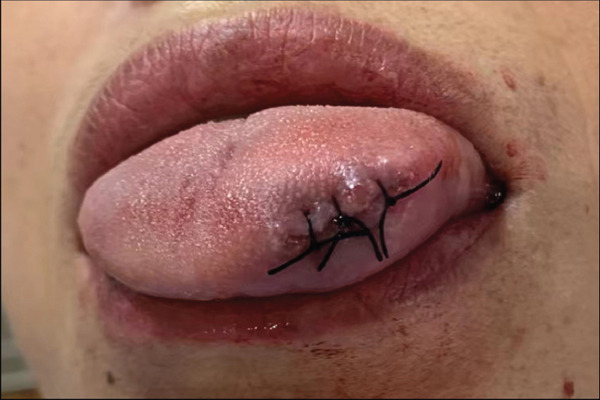
The surgical wound was closed with interrupted sutures using 3‐0 braided nonabsorbable silk sutures.

**Figure 6:**
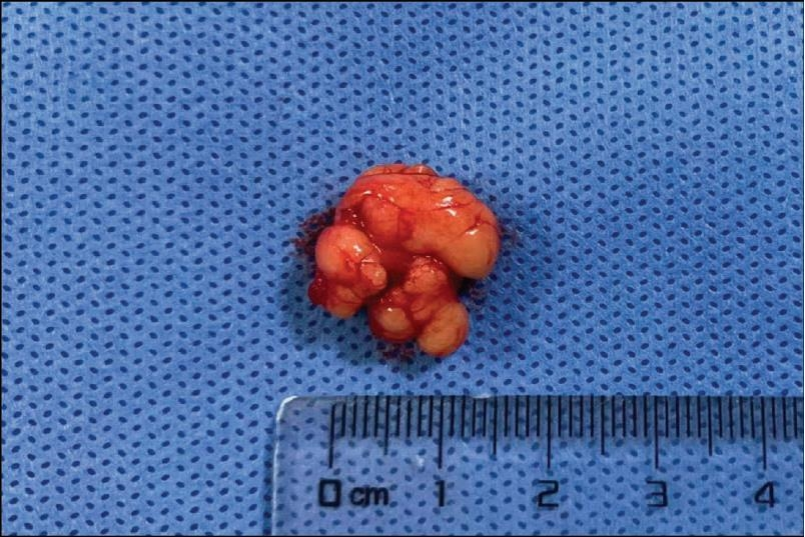
The excised lesion measured approximately 1.5 × 1.5 × 0.9 cm, yellowish in color, with an intact capsule and soft texture.

Routine preoperative evaluation ruled out surgical contraindications, and minimally invasive resection was performed under local anesthesia. A longitudinal incision was made along the highest mucosal bulge of the left lateral tongue with a No. 15 surgical blade. A pale yellow tumor mass protruded beneath the lingual mucosa after incision (Figure 3). Blunt dissection with mosquito forceps combined with sharp dissection was applied to completely resect the encapsulated lesion (Figure 4). The surgical wound was closed with interrupted 3‐0 silk sutures (Figure 5). The gross specimen was pale yellow and soft, with a size of approximately 1.5 × 0.9 cm (Figure 6).

### 2.5. Pathological Examination

**Figure 7:**
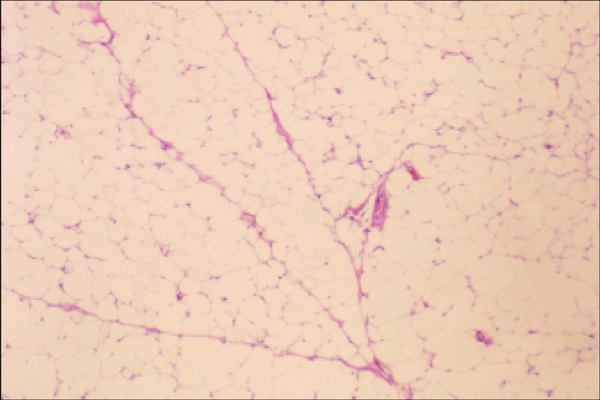
Histopathological examination (hematoxylin and eosin staining, light microscopy) showed that the tumor was mainly composed of mature adipocytes, without cellular atypia or mitotic figures, consistent with the pathological features of lipoma.

The excised specimen was fixed in formalin, dehydrated, embedded in paraffin, and stained with hematoxylin and eosin (HE). Histopathological examination showed mature adipocytes with uniform size, peripherally located nuclei, and regular arrangement, with delicate fibrovascular stroma, no cellular atypia, and no mitotic figures, consistent with a classic lipoma (Figure 7).

### 2.6. Timeline

The timeline of diagnosis and treatment is presented in Table 1.

**Table 1:** Timeline of diagnosis and treatment.

Timepoint	Key events
12 months before presentation	The patient incidentally detected a small painless submucosal lesion at the left lateral tongue. The mass enlarged slowly and progressively without any subjective symptoms, and no intervention was received.
On the day of consultation	Physical examination identified a pale yellow submucosal mass of approximately 1.5 cm in diameter on the left lateral tongue. Contrast‐enhanced maxillofacial CT was prescribed for further evaluation.
On the day of consultation	Contrast‐enhanced CT was performed. The lesion was preliminarily diagnosed as a cystic space‐occupying lesion, and a definite preoperative diagnosis of lipoma could not be established.
On the day of consultation	Routine preoperative laboratory tests were completed to rule out surgical contraindications, and minimally invasive resection under local anesthesia was formulated as the treatment plan.
On the day of consultation	Complete minimally invasive resection of the lingual mass was performed under local anesthesia. The excised specimen was fixed in formalin, embedded in paraffin and stained with hematoxylin and eosin (HE).
7 days postoperatively	The patient returned for suture removal. The surgical wound healed well without erythema, exudation or other infectious complications. Histopathological examination confirmed the diagnosis of lipoma.
3 weeks postoperatively	Follow‐up visit was conducted. The wound achieved complete healing with no evidence of tumor recurrence. Lingual movement, mastication and speech functions fully returned to normal.

### 2.7. Diagnostic Challenges

This case illustrates multiple factors contributing to the preoperative misdiagnosis of a small lateral tongue lipoma as a cystic lesion, including imaging limitations, patient‐related factors and unique anatomical features. Firstly, small lipomas lose characteristic hypodense imaging features of adipose tissue on CT scans. The radiological manifestations overlap considerably with those of oral mucous cysts and other cystic lesions, resulting in poor imaging specificity, which is the primary cause of misdiagnosis. Secondly, economic factors limited the selection of auxiliary examinations. Magnetic resonance imaging (MRI) is the optimal modality to differentiate adipocytic neoplasms from cysts due to its high specificity for fatty tissue. However, the patient declined MRI examination owing to its relatively high cost, and only contrast‐enhanced CT was performed, precluding a definitive diagnosis via multimodal imaging. Thirdly, special anatomical characteristics and insufficient clinical experience further led to diagnostic errors. The lateral tongue has sparse subcutaneous adipose tissue, so small lipomas at this site are extremely rare. Most clinicians have limited experience with such uncommon lesions, which eventually results in inaccurate preoperative assessment.

### 2.8. Follow‐Up and Outcomes

**Figure 8:**
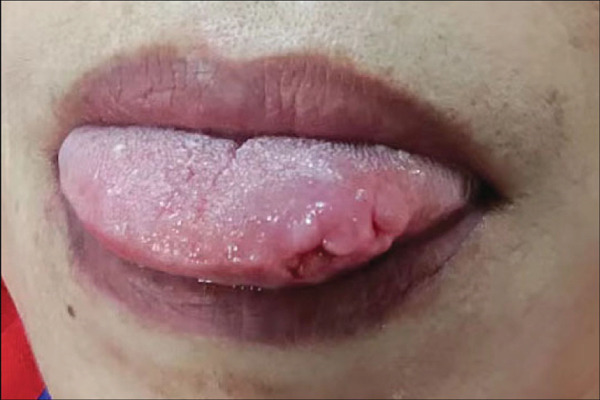
Sutures were removed on the 7th postoperative day.

**Figure 9:**
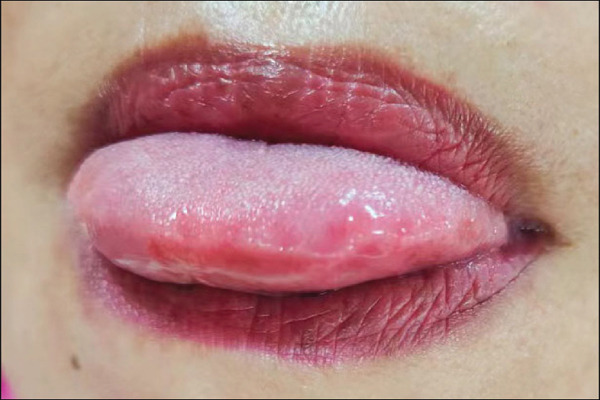
At 3 weeks postoperatively, the wound healed well, and the tongue morphology was satisfactorily restored.

Postoperative anti‐infective and symptomatic support treatment was administered. Sutures were removed on postoperative day 7 with uneventful wound healing (no bleeding, exudate, or infection) (Figure 8). At the 3‐week follow‐up, the surgical site healed completely, and no tumor recurrence was observed (Figure 9). The patient regained full function of tongue movement, swallowing, and speech. No pain or foreign body sensation was reported, and normal eating and verbal function were restored. The patient was satisfied with the surgical outcome.

## 3. Discussion

This 53‐year‐old female patient with a small lipoma on the lateral tongue is consistent with the epidemiological feature that oral lipomas predominantly occur in individuals aged over 40 years [11]. The lesion had a maximum diameter of 1.5 cm, a clinical course of 1 year, slow growth, and no functional impairment, which is in line with the general characteristics of most small oral lipomas. Such lesions are usually asymptomatic in the early stage and easily overlooked due to their small size, leading to delayed medical presentation [11].

The postoperative recurrence of lingual lipomas is closely associated with growth pattern, infiltration range, and integrity of surgical resection margins. As a special subtype, intramuscular lipoma grows in an infiltrative manner. Previous studies have reported its postoperative recurrence rate ranging from 3% to 62.5% [18]. In contrast, superficial lingual lipomas with intact capsules and well‐defined borders have a favorable prognosis, with a recurrence rate lower than 2% and almost no recurrence after complete excision [6]. Multiple factors contribute to the recurrence of lipomas after surgical removal. Firstly, infiltrative growth and incomplete capsules may leave tiny residual lesions within muscle spaces [19]. Secondly, the complex neurovascular anatomy of the tongue makes it difficult to achieve adequate surgical margins [20]. Thirdly, systemic metabolic disorders, including dyslipidemia, obesity, and insulin resistance, can induce abnormal proliferation of residual adipogenic progenitor cells [21]. The present lesion possessed a complete fibrous capsule and was confined locally without muscular infiltration. Complete enucleation along the capsule was performed intraoperatively, so the long‐term recurrence risk is theoretically extremely low. Nevertheless, long‐term follow‐up is still required for further verification.

Imaging examination plays a vital role in the preoperative diagnosis of lingual lipomas. Contrast‐enhanced CT can reveal characteristic hypodense manifestations of adipose tissue [16]. However, for small submucosal lingual lipomas, the partial volume effect averages the CT values due to the small lesion size and overlapping signals from surrounding mucosa and soft tissues, resulting in CT values close to those of cysts. Meanwhile, peripheral mucosal enhancement may obscure the fatty boundary, which frequently leads to a radiological diagnosis of cystic space‐occupying lesion [16]. Negative Hounsfield units (HU) are a typical imaging parameter of adipose tissue. Literature has documented that the CT values of simple lingual lipomas range from −134 to −83 HU [16]. In this case, the CT values of the lesion were measured from −48 to −8 HU, with an average value of approximately −24 HU, which was markedly higher than the reported range. The small lesion (maximum diameter: 1.5 cm) contained uniformly compact adipose tissue and a small number of delicate fibrous septa, without fat cords. Mild peripheral enhancement was observed, and some fibrous septa showed similar density to cystic walls. Disturbed by the partial volume effect, the typical CT features of fat were masked, thus causing preoperative misdiagnosis as a cyst. Clinically, lipomas larger than 2 cm present obvious fat stratification and typical HU values, with a remarkably lower misdiagnosis rate. For tiny submucosal lipomas, limited lesion size tends to conceal characteristic CT findings, which is the major cause of CT misdiagnosis [16].

MRI provides higher diagnostic specificity for lipomas. Characteristically, lipomas show hyperintensity on T1‐ and T2‐weighted sequences and hypointensity on fat‐suppressed sequences, which enables effective differentiation between adipose tissue and cystic lesions. Additionally, MRI carries no risk of ionizing radiation to patients, making it the optimal imaging modality for cases with suspected lipomas [22]. Nevertheless, CT remains the mainstream examination in general and primary hospitals because of its low cost, rapid scanning, and convenient operation [16]. Based on the clinical experience of this case, combining typical physical signs (soft texture, good mobility, and pale yellow submucosal appearance) with contrast‐enhanced CT findings can effectively differentiate lipomas from other benign cystic lesions and improve the preoperative diagnostic accuracy for small lateral tongue lipomas. This combined diagnostic strategy is practical and suitable for primary medical institutions.

A variety of benign lesions may manifest as protruding masses on the lateral tongue. Differential diagnoses mainly include epidermoid cysts, mucous cysts, fibromas, well‐differentiated liposarcomas, and angiolipomas [16], which can be differentiated based on the following features:

1. Epidermoid cysts: These lesions are relatively firm on palpation and filled with keratin debris. Their CT values range from +20 to +40 HU, without negative HU values. Only cystic walls show enhancement on contrast scans. By comparison, lipomas are soft and present characteristic negative fatty density on CT.
2. Lingual mucous cysts: They predominantly occur on the ventral tongue and floor of the mouth, and are often associated with a history of biting. The lesions appear translucent pale blue with homogeneous water‐like density (0–15 HU) on CT, and no signal attenuation is detected on fat‐suppressed MRI sequences due to the absence of adipose components.
3. Fibromas: They are solid dense soft‐tissue masses with CT values above +30 HU and intermediate signal on T1WI, showing no typical fatty signals.
4. Well‐differentiated liposarcomas: Such lesions are usually larger than 4 cm with ill‐defined borders and infiltration into adjacent muscles. Mixed high and low HU values and irregular solid nodules can be observed inside, and the lesions progress rapidly. Atypical cellular features can be identified on pathological examination. This case had a small size, clear boundary, and no cellular atypia, so liposarcoma was excluded.
5. Angiolipomas: Characterized by numerous tiny vascular cords and obvious heterogeneous enhancement after contrast administration. The present lesion only showed mild peripheral enhancement without abundant vascular components, inconsistent with the features of angiolipoma.

Complete surgical excision is the first‐line treatment for oral lipomas, associated with favorable long‐term outcomes and a very low recurrence rate [23, 24]. Minimally invasive resection under local anesthesia was performed in this case, which proved to be safe and effective. This surgical approach has multiple advantages. First, a longitudinal incision following the anatomical course of the tongue minimizes trauma to lingual muscles and nerves and preserves normal lingual function. Second, combined blunt and sharp dissection enables complete removal of the encapsulated mass and reduces recurrence risk. Third, local anesthesia avoids general anesthesia‐related complications, with the advantages of minimal trauma, low cost, and rapid postoperative recovery. The satisfactory postoperative recovery of this patient further confirms that this minimally invasive procedure is the optimal option for such small lesions.

Most existing literature focuses on moderate and giant lipomas located on the dorsum, ventral tongue, buccal mucosa, and lips [25–29]. Reports regarding small lipomas on the lateral tongue are extremely scarce, which constitutes the core innovation and clinical value of the present study. Firstly, this article comprehensively documents the full clinical data, detailed CT parameters, surgical procedures and pathological results of this rare small lipoma at an uncommon lingual site. Secondly, it elaborates the imaging mechanism of CT misdiagnosis caused by atypical CT values of small lipomas, providing valuable imaging references for primary care settings. Thirdly, this case verifies that small lipomas on the tongue margin can be completely resected via minimally invasive small incisions under local anesthesia, establishing a feasible minimally invasive treatment protocol for such rare small lesions and supplementing the current literature dominated by moderate and giant lingual lipomas. In summary, this rare small lipoma of the lateral tongue enriches the clinical dataset of this unusual subtype and provides references for individualized diagnosis and treatment of similar lesions. The core indicators of this case were compared with those of recently published cases of lingual lipomas (Table 2).

**Table 2:** Comparison of clinical features between the present case and previously reported lingual lipoma cases.

Clinical features	Lingual lipomas reported in previous literature	Present case
Age at onset	Predominantly occurs in middle‐aged and elderly individuals aged 40–65 years, with a mean age of 51.2 years.	The patient was 53 years old (within the high‐incidence age group), whereas the lesion was remarkably small in size.
Maximum tumor diameter	Mean diameter of 3.0 cm; most lesions are larger than 2.0 cm, and giant lipomas can exceed 5 cm.	1.5 cm, far below the literature‐reported average, classified as a miniature lipoma at the lingual margin.
Lesion location	Mainly found on the dorsum, ventral surface of the tongue and floor of the mouth; lesions at the lingual margin are rare.	Left lingual margin (a clinically uncommon site).
Preoperative imaging	Lipomas larger than 2 cm show typical fat density on CT (characteristic Hounsfield units), with rare misdiagnosis as cysts.	The tiny lesion was misdiagnosed as a cystic lesion on CT due to volume averaging artifact, representing an atypical misdiagnosis case.
Surgical approach	Large tumors generally require extensive incisions, and general anesthesia is adopted in some cases.	Minimally invasive longitudinal incision under local anesthesia with combined blunt and sharp dissection, resulting in less surgical trauma.
Clinical symptoms	Large lesions are accompanied by impaired tongue movement and eating dysfunction.	The patient was completely asymptomatic, and the lesion was detected incidentally during physical examination.

The comparative results revealed that the average maximum diameter of lingual lipomas in previous reports was 3.0 cm, and some giant lesions exceeded 5 cm. Most lesions were located on the tongue dorsum, ventral surface, and floor of the mouth, and large masses frequently led to lingual movement disorders, dysphagia, and dysarthria. In contrast, the lesion in our patient was merely 1.5 cm in diameter, located at the rare lateral tongue site, and the patient had no subjective symptoms throughout the disease course.

Several limitations of this study should be acknowledged. Firstly, the pathogenesis was not fully explored. Accumulated evidence indicates that the development of oral lipomas is related to genetic predisposition, lipid metabolism disorders, chronic inflammation, lifestyle alterations, and hormonal changes [29–32]. Genetic mutations greatly increase the risk of familial lipomatosis [30]. Long‐term high‐fat diet, alcohol abuse, and obesity can induce lipid metabolic disorders and further trigger abnormal proliferation of adipocytes [33]. Local chronic inflammation and repeated trauma can also cause aberrant differentiation of adipocytes [34]. The patient had no definite history of trauma, chronic inflammation, or endocrine disorders, and no genetic testing was conducted. Therefore, the individual pathogenic factor could not be identified, and the underlying mechanism remains to be further investigated. Secondly, the follow‐up period was relatively short. Only 3‐week short‐term follow‐up was completed, which can merely confirm the short‐term efficacy and safety of the surgery. Long‐term prognosis and late tumor recurrence cannot be fully evaluated. We will continue to conduct long‐term follow‐up for at least 2 years, including clinical examinations at 6 months, 1 year, and 2 years postoperatively, and imaging tests will be arranged when necessary to supplement long‐term follow‐up data. Thirdly, this is a single‐case study with a limited sample size. Compared with large‐sample clinical trials, its generalizability and clinical guiding value are relatively restricted.

## 4. Conclusion

Small lipomas arising from the lateral tongue represent a rare type of benign mesenchymal neoplasm in the oral cavity. These lesions progress slowly and are mostly asymptomatic at the early stage, which leads to a high rate of clinical missed diagnosis and preoperative misdiagnosis as cystic masses. Combining characteristic physical signs with contrast‐enhanced CT findings serves as an effective strategy to improve the preoperative diagnostic efficacy for this disease. Minimally invasive resection via a longitudinal lingual incision under local anesthesia, with the application of combined blunt and sharp dissection, enables complete tumor removal while preserving physiological functions including tongue movement and sensation. This surgical approach yields a favorable postoperative outcome with a low recurrence risk. The present case report supplements the clinical data of tiny lipomas located at the lateral tongue. Nevertheless, this study has limitations due to its single‐center and single‐case design, so the research conclusions have limited generalizability. Further multicenter clinical studies with large sample sizes and long‐term follow‐up are required to continuously verify the reliability of the current diagnostic and therapeutic protocols. In addition, in‐depth exploration of the etiological factors and pathogenesis of oral lipomas is warranted, so as to provide theoretical support for disease prevention, control and individualized treatment.

## Author Contributions

**Guojun Yang** took charge of clinical practice and project administration, as well as manuscript review, revision, and overall study supervision. **Zhen Liu** completed data curation and resource arrangement. **Jingbiao Wu** was responsible for figure preparation and coreviewed and revised the manuscript. **Ming Zhang** wrote the original draft. **Qiao Li** collated pathological data and participated in the investigation. **Binbin Jing** performed literature retrieval and clinical data collection.

## Funding

No funding was received for this manuscript.

## Disclosure

All authors have read and approved the final version of the manuscript. The corresponding author had full access to all of the data in this study and takes complete responsibility for the integrity of the data and the accuracy of the data analysis.

## Ethics Statement

This is an anonymized single case report. Written informed consent was obtained from the patient prior to publication, so formal ethical approval was not required.

## Consent

Written informed consent has been secured from the patient prior to publication of this case report and accompanying imagery.

## Conflicts of Interest

The authors declare no conflicts of interest.

## Data Availability

Data sharing is not applicable to this article as no datasets were generated or analyzed during the current study.
